# Differential branch pulmonary artery regurgitant fraction identifies patients with unilateral stenosis associated with relatively elevated pulmonary vascular resistance in the contralateral lung after repair of conotruncal anomalies

**DOI:** 10.1186/1532-429X-11-S1-P116

**Published:** 2009-01-28

**Authors:** Matthew A Harris, Kevin K Whitehead, Matthew J Gillespie, Timothy I Liu, Michael T Cosulich, Paul M Weinberg, Mark A Fogel

**Affiliations:** The Children's Hospital of Philadelphia, Philadelphia, PA USA

**Keywords:** Public Health, Retrospective Review, Cardiac Magnetic Resonance, Size Discrepancy, Screening Tool

## Introduction

Repaired conotruncal anomaly pts may have residual branch pulmonary artery (BPA) stenosis or significant differences in the sizes of the BPAs. Phase-Contrast Magnetic Resonance (PCMR) can measure differential BPA regurgitation.

## Purpose

To determine if: (1) residual BPA stenosis or size discrepancy is associated with differential BPA regurgitation (2) differential BPA regurgitation correlates with differential pulmonary vascular resistance (PVR).

## Methods

We retrospectively reviewed 71 consecutive cardiac magnetic resonance (CMR) studies for BPA size and PCMR data. We also reviewed 13 consecutive pts who underwent both CMR and catheterization.

## Results

**Figure 1:**
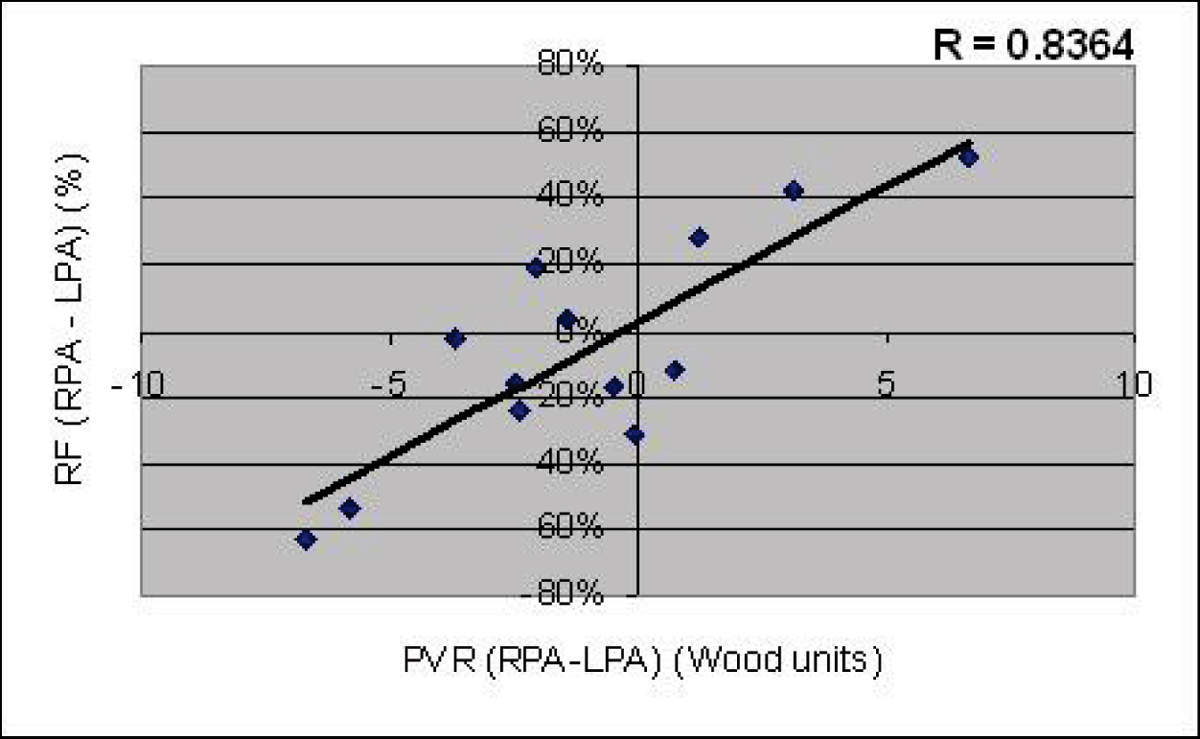
Figure 1

27 of the 71 pts had either BPA stenosis or one BPA cross-sectional area (CSA) comprise <33% of the total BPA CSA. Among these 27 pts, there was no significant difference between RPA and LPA regurgitant fraction (RF) (28 vs 32%, p = 0.49), however, there was a significantly increased RF of the larger vs smaller BPA (39 vs 21%, p < 0.001). In contrast, the 44 pts without BPA stenosis or size discrepancy showed a significant difference between RPA and LPA RF (31 vs 38%, p < 0.001), without a significant difference in the larger vs smaller BPA RF (36 vs 33%, p = 0.14). Retrospective review of pts who underwent both CMR and catheterization demonstrates that differential BPA RF strongly correlates with differential PVR (R = 0.8364, p < 0.001). See Figure 1.

## Conclusion

BPA stenosis or size discrepancy outweighs the increased LPA RF of pts without stenosis or size discrepancy. Since differential BPA RF correlates with differential PVR, PCMR can serve as an important screening tool for identifying pts with stenosis or size discrepancy who may have developed relatively increased PVR in the contralateral larger pulmonary artery.

